# Reduction of *Salmonella* Typhimurium Cecal Colonisation and Improvement of Intestinal Health in Broilers Supplemented with Fermented Defatted ‘Alperujo’, an Olive Oil By-Product

**DOI:** 10.3390/ani10101931

**Published:** 2020-10-21

**Authors:** Agustín Rebollada-Merino, María Ugarte-Ruiz, Marta Hernández, Pedro Miguela-Villoldo, David Abad, David Rodríguez-Lázaro, Lucía de Juan, Lucas Domínguez, Antonio Rodríguez-Bertos

**Affiliations:** 1 VISAVET Health Surveillance Centre, Complutense University of Madrid, 28040 Madrid, Spain; agusrebo@ucm.es (A.R.-M.); pedromig@ucm.es (P.M.-V.); dejuan@visavet.ucm.es (L.d.J.); lucasdo@visavet.ucm.es (L.D.); arbertos@ucm.es (A.R.-B.); 2 Department of Internal Medicine and Animal Surgery, Faculty of Veterinary Medicine, Complutense University of Madrid, 28040 Madrid, Spain; 3 Laboratorio de Biología Molecular y Microbiología, Instituto Tecnológico Agrario de Castilla y León, 47071 Valladolid, Spain; ita-HerPerMa@itacyl.es (M.H.); abagarda@itacyl.es (D.A.); drlazaro@ubu.es (D.R.-L.); 4 Área de Microbiología, Departamento de Biotecnología y Ciencia de los Alimentos, Universidad de Burgos, 09001 Burgos, Spain; 5 Department of Animal Health, Faculty of Veterinary Medicine, Complutense University of Madrid, 28040 Madrid, Spain

**Keywords:** antimicrobial alternatives, fermented defatted ‘alperujo’, intestinal health, olive oil by-products, *Salmonella* Typhimurium

## Abstract

**Simple Summary:**

*Salmonella* spp. is a bacterium that places human health at risk by consuming eggs and poultry. In the European Union, the use of antimicrobials to treat salmonellosis in aviculture is no longer permitted due to the resistance to treatment of some bacteria, such as *Salmonella* spp. For this reason, compounds derived from natural food sources are being increasingly tested to assess their efficacy against *Salmonella* spp. In this study, chickens were given dietary supplements in the form of fermented defatted ‘alperujo’, a modified olive oil by-product, after which they were infected with *Salmonella* Typhimurium. The chickens given the supplement showed a healthy gut and a reduction in the amount of *Salmonella* spp. in the cecum. In conclusion, this olive oil by-product may contribute to preventing and controlling salmonellosis in farms, as well as reducing environmental contamination.

**Abstract:**

*Salmonella* spp. contaminates egg and poultry meat leading to foodborne infections in humans. The emergence of antimicrobial-resistant strains has limited the use of antimicrobials. We aimed to determine the effects of the food supplement, fermented defatted ‘alperujo’ (FDA), a modified olive oil by-product, on *Salmonella* Typhimurium colonisation in broilers. One hundred and twenty 1-day-old broilers were divided into four experimental groups—two control groups and two treated groups, and challenged with *S*. Typhimurium at day 7 or 21. On days 7, 14, 21, 28, 35, and 42 of life, duodenum and cecum tissue samples were collected for histopathological and histomorphometric studies. Additionally, cecum content was collected for *Salmonella* spp. detection by culture and qPCR, and for metagenomic analysis. Our results showed a significant reduction of *Salmonella* spp. in the cecum of 42-day-old broilers, suggesting that fermented defatted ‘alperujo’ limits *Salmonella* Typhimurium colonization in that cecum and may contribute to diminishing the risk of carcass contamination at the time of slaughter. The improvement of the mucosal integrity, observed histologically and morphometrically, may contribute to enhancing intestinal health and to limiting *Salmonella* spp. colonisation in the host, mitigating production losses. These results could provide evidence that FDA would contribute to prophylactic and therapeutic measures to reduce salmonellosis prevalence in poultry farms.

## 1. Introduction

*Salmonella* spp. is Gram-negative intracellular enteric bacterium of public health concern. It is responsible for over 90,000 cases of zoonotic food-borne infections per year in the European Union (EU), according to the last European Food and Safety Authority (EFSA) report [1]. In recent years, different serovars were considered responsible for disease outbreaks, and *Salmonella* Typhimurium and *S*. Enteritidis were the most prevalent in the European Union [1].

The main source of human infections in high-income countries is associated with the consumption of eggs and poultry meat, which usually become contaminated during the slaughtering process through the food chain [1]. Salmonellosis in poultry causes decreased growth and eventual mortality in chickens [2], although normally chickens are asymptomatic carriers and shed the bacteria in their feces [3,4]. Due to the role of poultry in disseminating *Salmonella* spp., control measures, such as those included in National Control Plans, have been implemented in the EU to reduce the prevalence of salmonellosis and other foodborne diseases in poultry production [5].

The emergence of antimicrobial-resistant strains limits the use of antimicrobials to treat *Salmonella* spp. infections in poultry in the EU [2,3,6]. For this reason, the complementary use of compounds with antimicrobial proprieties as feed additives or supplements has been promoted [3]. These supplements include probiotic, prebiotic, phytobiotic, and nutraceutical products [7,8].

During the olive oil two-phase extraction system, a semisolid by-product known as two-phase mill waste, olive pomace, or ‘alperujo’ is obtained [9]. The olives contain phenolic compounds that are retained in olive oil and in the by-products generated during olive oil processing [10], like olive pomace [11]. In vitro, olive oil by-products stimulate the synthesis of metabolites with bactericidal proprieties, and immune response [7,8,12]. In particular, oleuropein, decarboxymethyl ligstroside tyroxol, and hydroxytyrosol are known to possess antimicrobial proprieties [8,10,12,13], which could help to limit pathogen bacterial infection, colonisation, and excretion. Olive oil extracts were said to inhibit the growth of *Salmonella* Typhimurium and *S.* Enteritidis in vitro [8,10,12,13]. Besides, in broilers, olive pomace extract has demonstrated anti-inflammatory proprieties [11]. However, mass application of these extracts in animal production is difficult and costly.

In broilers and laying hens, direct supplementation of fermented defatted ‘alperujo’ (FDA) has been shown to improve intestinal morphology and induce changes in the microbiota [14,15], suggesting a beneficial effect on intestinal health. The improvement in the response of intestinal mucosa to eventual damage may lead to controlling *Salmonella* spp. colonisation in the gut and thus limiting transmission within the farm and through the food chain [16]. However, the effects of FDA supplementation on controlling pathogenic bacteria as *Salmonella* spp. in the poultry gut has not yet been investigated.

The aim of this study was to evaluate the effects that FDA has on the intestinal mucosal morphology (duodenum and cecum) and on cecal reduction of *Salmonella* Typhimurium in broilers infected with *Salmonella* Typhimurium at 7 or 21 days old. We assumed that the antimicrobial proprieties previously described in olive oil by-products could contribute to mitigating *Salmonella* spp. infection in broilers.

## 2. Materials and Methods

### 2.1. Ethical Approval and Animal Welfare

All the experimental procedures were approved by the Animal Care and Ethics Committee of the Complutense University of Madrid in compliance with the Community of Madrid (PROEX 152/19). Animal experiments took place in the biosafety level 3 (BSL-3) facilities of the VISAVET Health Surveillance Centre. One-day-old male Ross 308 broilers were housed according to the European legislation on animal welfare (Directive 2010/63/EU): water and food were provided ad libitum, and temperature and light/dark cycles controlled according to age.

### 2.2. Animal Groups and Feed

The distribution of the experiment and the animals included two separate boxes with two cages each—one treated group and one control group per box, with 30 animals per cage (*n* = 120 animals). Controls were fed with conventional broiler feed. Since their arrival, treated animals received the same commercial feed as the control group but were given a supplement of 2% FDA as previously described [14,15]. FDA composition is detailed in Table 1 [14].

### 2.3. Salmonella Typhimurium Challenge

Immediately after arrival, animals were tested for *Salmonella* spp. following ISO 6579-1:2017 [17], obtaining negative results. After 3 h of water restriction, at day 7 or 21, all animals in boxes 1 or 2, respectively, were challenged with 3.3 × 10^5^ CFU/mL of monophasic variant of colistin-resistant *S*. Typhimurium (mcr-1 positive) suspended in a total volume of 330 mL of drinking water. A clinical examination was performed twice-a-day upon arrival and at the end of the experiment, with special focus on water and food consumption, animal welfare, emergence of clinical signs after the *Salmonella* Typhimurium challenge, and eventual mortality.

### 2.4. Postmortem Examination and Samplings

Five randomly-selected animals from each group (*n* = 20) were sedated with diazepam (1 mg/kg intramuscular) and euthanised with an overdose of sodium pentobarbital (100 mg/kg intravenous) on days 7, 14, 21, 28, 35, and 42 of life. A complete post-mortem survey was performed for each animal. Cecum feces were collected in parallel for *Salmonella* spp. detection using traditional culture and conserved at −80 °C for molecular analysis and further metagenomic studies. Duodenum and cecum tissue samples were fixed in 10% of commercial buffered formaldehyde solution.

### 2.5. Salmonella *spp.* Detection and Culture Conditions

*Salmonella* spp. detection was performed following a protocol already published [18], although samples were streaked onto SMID2 agar plates (BioMérieux, Marcy-l’Étoile, France) instead of ColR agar plates (BioMérieux), using serial dilutions in order to quantify the presence of this *Salmonella* spp. The agar plates were examined after incubation at 37 °C for 24 h under aerobic conditions. All colonies resembling *Salmonella* spp., according to the manufacturer’s instructions, were counted.

### 2.6. Real-Time PCR for Salmonella *spp.* Detection

The detection of *Salmonella* spp. by real-time PCR (qPCR) was based on previously-described assays [19,20], using 3 μL of the same total cecal DNA extracted for 16S rRNA Library Preparation and sequencing as a template. Reactions were run on an Applied BioSystems 7500 (Applied BioSystems, Foster City, CA, USA). Amplification was performed using an initial hot-start step at 95 °C for 10 min, followed by 40 cycles of a denaturation step at 95 °C for 30 s and an annealing/extension step at 55 °C for 60 s. Fluorescence was only recorded at the end of the annealing/extension step. Three qPCR replicates were used for each sample.

### 2.7. 16S rRNA Library Preparation and Sequencing

Total DNA was extracted from 220 mg of cecal content using a commercial kit (QIAamp DNA Stool Mini Kit, Qiagen, Hilden, Germany) and DNA concentration was determined using a fluorometer (Qubit fluorometer, Invitrogen, Carlsbad, CA, USA). Microbial diversity was assessed by analyzing sequences of the V3–V4 region of the 16S rRNA gene. The primers and PCR conditions used for this analysis were as previously reported [21]. Sample multiplexing, library purification, and sequencing were performed as described in the “16S Metagenomic Sequencing Library Preparation” guide by Illumina (San Diego, CA, USA). Libraries were sequenced on an Illumina MiSeq platform that provided 300-bp paired-end reads.

### 2.8. Bioinformatics and Data Analysis

Raw demultiplexed sequence data was processed using QiimeReporter (https://github.com/dabadgarcia/qiimereporter, Instituto Tecnologico Agrario de Castilla y Leon, Valladolid, Spain). This straightforward pipeline for the analysis of amplicon sequences integrates basic Qiime2 commands [22] with the R programming language. In brief, the DADA2 package [23] was used to filter reads, merge paired ends, remove chimeras, and assign amplicon sequence variants (ASV). Then, a pre-trained Naïve Bayes classifier [24] was used to obtain the taxonomic assignment of the ASVs, using SILVA database version 132 (SILVA ribosomal RNA database project, Bremen, Germany) as a reference [25], which resulted in a table containing the microbial composition for each of the samples. Raw reads are available in the BioProject database with ID PRJNA643396, samples SAMN16203983 to SAMN16203863 (http://www.ncbi.nlm.nih.gov/bioproject/).

### 2.9. Histological Processing, and Histopathological and Histomorphometric Analysis

After fixation, routine histological processing and hematoxylin–eosin staining was carried out as described elsewhere [14,15]. A histopathological study focused on morphological features (villous stunting in duodenum, epithelial injury, crypt hyperplasia, crypt distortion, lamina propria oedema, lacteal dilation, mucosal fibrosis, and hemorrhages) and inflammation (intraepithelial lymphocytes, lamina propria lymphocytes, heterophils, eosinophilic granular cells, and/or macrophages, and mucosal-associated lymphoid tissue) was performed at 7 and 14 days post-infection (dpi). In addition, samples of duodenum and cecum were subjected to a histomorphometric analysis at 400× magnifications employing an image analyzer (Leica Application Suite, Leica, Wetzlar, Germany), as described elsewhere [14,15]. Twenty intact and well-oriented villi in duodenum and 20 crypts in duodenum and cecum were measured in each animal. Duodenum villi were measured from the top to the crypt–villus junction. Crypts were measured from the crypt–villus junction in the duodenum or the mucosal surface in the cecum to the basement membrane.

### 2.10. Statistical Analysis

A statistical analysis of *Salmonella* spp. culture, qPCR, and histomorphometry was performed using Mann–Whitney and Fisher exact tests following IBM SPSS Statistics Software v25 (IBM, Armonk, NY, USA). The level for statistical significance was set at *p* < 0.05.

## 3. Results

### 3.1. Clinical Signs and Gross Findings

During the experiment, no clinical signs or mortality were reported. The post-mortem examination revealed mild diffuse chronic catarrhal enteritis in animals belonging to the control group. Occasional hepatic congestion or steatosis, not associated with treatment or challenge age, was also reported.

### 3.2. Salmonella Typhimurium Colonisation in the Cecum

**Figure 1:**
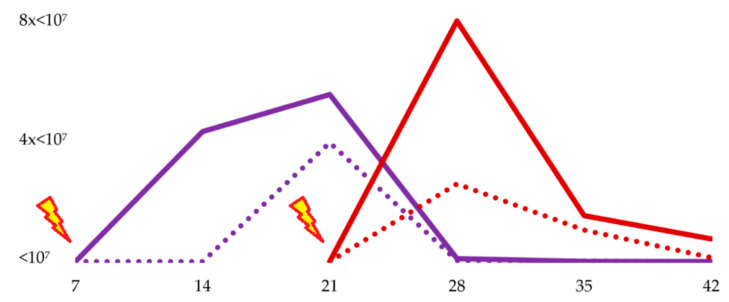
Results of the *Salmonella* spp. count in selective agar in broilers challenged with *Salmonella* Typhimurium at day 7 (purple lines) and day 21 (red lines) of life. Continuous lines represent control groups, whereas discontinuous lines represent treated groups, fed with fermented defatted ‘alperujo’. The *Salmonella* spp. count by culture (CFU/g) is shown on the vertical axis, and samplings (7, 14, 21, 35, or 42) on the horizontal axis.

**Figure 2:**
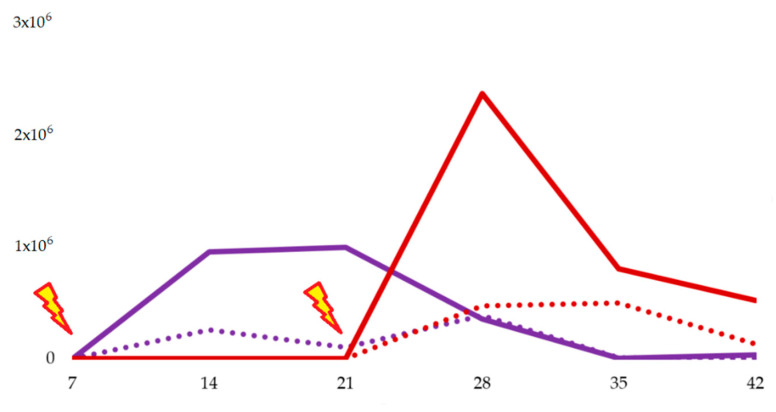
Results of the qPCR for *Salmonella* spp. in broilers challenged with *Salmonella* Typhimurium at day 7 (purple lines) and day 21 (red lines) of life. Continuous lines represent control groups, whereas discontinuous lines represent treated groups, fed with fermented defatted ‘alperujo’. The genome equivalent/g is shown on the vertical axis, and samplings (7, 14, 21, 35, or 42) on the horizontal axis.

In 7-day-old challenged chickens, at 7 dpi (14 days old), the load of *Salmonella* spp. in the cecum was significantly lower than controls by culture (*p* = 0.008), and almost by qPCR (*p* = 0.056). At 14 dpi (21 days old), there were significant differences in the *Salmonella* spp. cecal load between the control and treated group by qPCR (*p* = 0.032) but not by culture (*p* > 0.05). At 21, 28, and 35 dpi (28, 35, and 42 days old, respectively) there were no significant differences in the cecal *Salmonella* spp. load among groups by culture (*p* > 0.05) or by qPCR (*p* > 0.05). (Figure 1 and Figure 2, Table 2).

In 21-day-old challenged broilers, all cecal content challenged was negative for *Salmonella* spp. either by culture or qPCR. At 7 dpi (28 days old), the *Salmonella* spp. load in the cecum was significantly reduced in the treated group by qPCR (*p* = 0.016), and by culture, which was not significant (*p* = 0.075). At 14 dpi (35 days old), there were no significant differences in the cecal *Salmonella* spp. load among groups by culture (*p* > 0.05), or by qPCR (*p* > 0.05). Finally, at 21 dpi (42 days old) there was a significant reduction in treated broilers by qPCR (*p* = 0.016), not by culture (*p* = 0.076) (Figure 1 and Figure 2, Table 3).

The number of positive chickens by culture and qPCR at both challenge times is detailed in Table 4.

### 3.3. Intestinal Histopathology

**Figure 3:**
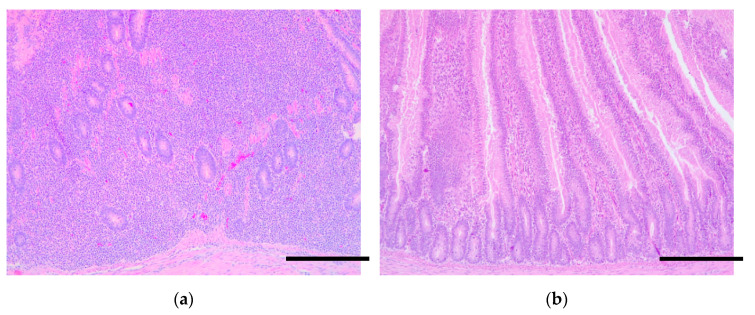
Histopathological study of the duodenum of 14-day-old broilers, 7 days post-infection with *Salmonella* Typhimurium. (**a**) Control group. There is a severe lymphocytic inflammatory infiltrate that completely distorted the crypt structure. There are scattered haemorrhagic foci. (**b**) Treated group. The lamina propria is mildly expanded by a lymphocytic inflammatory infiltrate with a few heterophils. 20×, scale bar: 250 µm.

In both control groups, in 7- or 21-day-old challenge, the duodenum at 7 dpi (14 or 28 days of life) displayed a moderate atrophy and stunting of the villi with mild epithelial desquamation and a slight increase in intraepithelial lymphocytes. The lamina propria was moderately to severely expanded by an inflammatory infiltrate composed of lymphocytes, heterophils, and macrophages that partially distorted the crypt structure (Figure 3a). At 14 dpi (21 or 35 days of life), in the control group the lesions were similar, with additional mild crypt distortion and mild gut-associated lymphoid tissue (GALT) hyperplasia. In the treated group, there was a reduction in the severity of villous stunting and lymphocytic infiltrate (Figure 3b).

**Figure 4:**
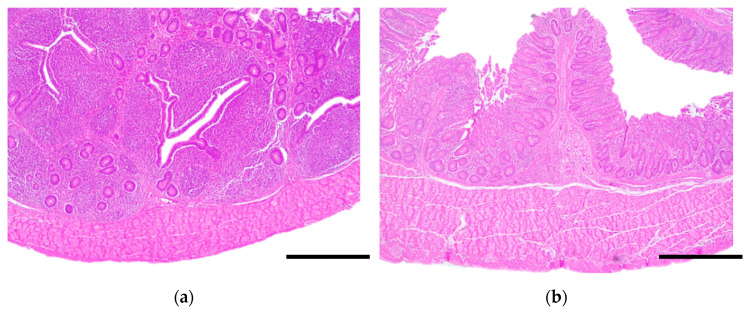
Histopathological study of the cecum of 14-day-old broilers, 7 days post-infection with *Salmonella* Typhimurium. (**a**) Control group. There is a severe lymphocytic inflammatory infiltrate that completely distorted the crypt structure. GALT hyperplasia is evident. (**b**) Treated group. The lamina propria is mildly expanded by a lymphocytic inflammatory infiltrate. 20×, scale bar: 250 µm.

In the cecum, at 7 dpi (14 or 28 days of life), control groups presented a mild epithelial desquamation and the lamina propria was slightly expanded by an infiltrate composed of lymphocytes and plasma cells that moderately to severely distorted the crypt structure. GALT hyperplasia was moderate to severe (Figure 4a). By 14 dpi (21 or 35 days of life), all those changes were maintained in both control groups with an additional increase in intraepithelial lymphocytes. In the treated group, there was a reduction in the intensity of lamina propria lymphocytic infiltration and GALT hyperplasia compared to the control group (Figure 4b).

### 3.4. Intestinal Morphology

In 7-day-old challenged chickens, duodenum villi height was significantly improved in treated chickens on days 7, 14, 28, 35, and 42 (*p* < 0.05). Similarly, the crypts in the duodenum were deeper in in all treated samplings (*p* < 0.05). Regarding ceca morphology, crypts were seen to be deeper in 7-, 21- and 42-day-old treated chickens (*p* < 0.05). At 28 days of life, controls displayed a higher value for crypt depth (*p* < 0.05) (Table 5).

Broilers in the treated group challenged at 21 days of age showed a significant improvement in the duodenum villi height at 28, 35, and 42 days of life (*p* < 0.05). The depth of the crypts in the duodenum was significantly improved by the treatment on days 28 and 42 (*p* < 0.05). The cecum crypt was deeper in treated chickens on days 35 and 42 of life (*p* < 0.05) (Table 5).

### 3.5. Cecal Microbiota

**Figure 5:**
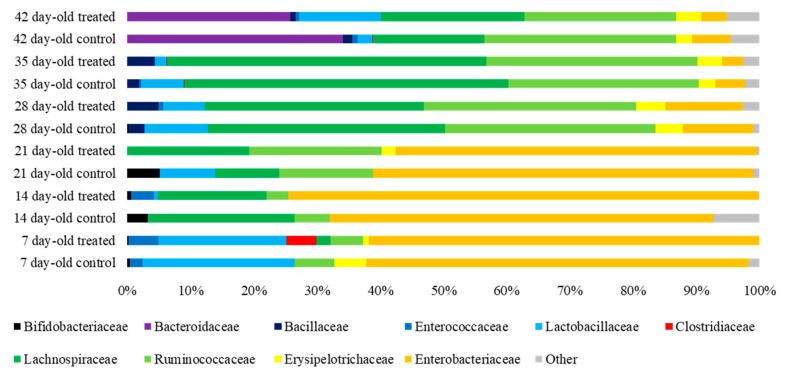
Bar chart showing the 10 most abundant bacterial families found in the cecal content of control and treated broiler chickens challenged with *Salmonella* Typhimurium at 7 days of life. Each bar represents the relative abundance (horizontal axis) of bacterial families by group of animals, diet treatment, and age.

In chickens challenged at 7 days of age, there were no statistically-significant differences among the groups established. The most abundant bacterial family at days 7, 14, and 21 of life was *Enterobacteriaceae* in both groups. At day 28, *Enterobacteriaceae* drastically decreased and was replaced by *Lachnospiraceae* and *Ruminococcaceae* in similar abundance, and these were also the most prevalent families at 35 days of life, with a higher abundance of *Lachnospiraceae*. Finally, at day 42 of life, *Bacteroidaceae*, *Ruminococcaceae,* and *Lachnospiraceae* were the most prevalent families in both groups (Figure 5).

**Figure 6:**
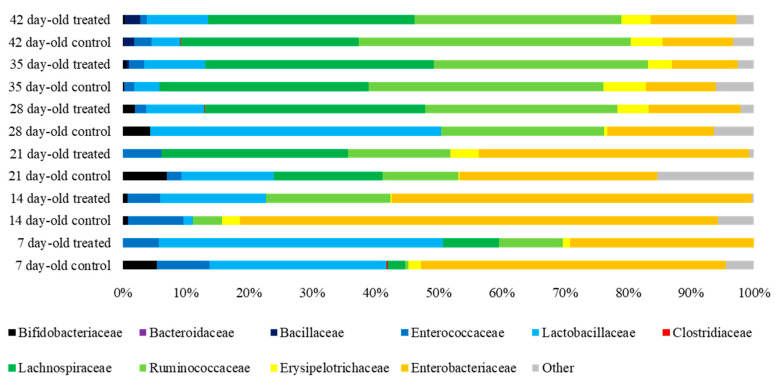
Bar chart showing the 10 most abundant bacterial families found in the cecal content of control and treated broiler chickens challenged with *Salmonella* Typhimurium at 21 days of life. Each bar represents the relative abundance (horizontal axis) of bacterial families by group of animals, diet treatment, and age.

In chickens challenged at 21 days of age, there were no statistically-significant differences among the groups established. The most abundant bacterial family at day 7 was *Enterobacteriaceae* in the control group and *Lactobacillaceae* in the treated group. At day 14 of life, *Enterobacteriaceae* was still prevalent in the control group, whereas in the treated group *Enterobacteriaceae* and *Ruminococcaceae* were more abundant, being substituted by *Lachnospiraceae* as the second-most-abundant family in both groups at 21 days of life. In 28-day-old broilers, *Lactobacillaceae* and *Ruminococcaceae* were the most abundant families in the control group, whereas *Lachnospiraceae* and *Ruminococcaceae* were predominant in treated chickens. On days 35 and 42 of life, *Ruminococcaceae* and *Lachnospiraceae* were the most abundant families, with slightly higher values of *Ruminococcaceae* over *Lachnospiraceae* in the control groups (Figure 6).

## 4. Discussion

A reduction of the intestinal colonisation of *Salmonella* spp. in broilers may contribute to reducing bacterial shedding in the environment, thus avoiding transmission in the farm and contamination of poultry products through the food chain [26,27]. *Salmonella* spp. infection in chickens usually occurs in the early stages due to their impaired immunity [7,28,29]. We have proposed an infection in 7- or 21-day-old chickens with weekly samplings to evaluate the dynamics of infection in control and treated animals. The cecum is known to be the main site of colonisation for *Salmonella* spp. [27,28], and therefore fresh feces were collected from this segment for analysis. Culture- and qPCR-positive results confirmed that a *Salmonella* Typhimurium infection was established. The control group showed an initial increase in the *Salmonella* spp. count in the cecum until 14 dpi, followed by a rapid decrease, as expected according to previous studies [7,27,28,29]. Other authors observed a high rate of persistent infection in older chickens infected in the first week of life [30], although this could be explained by the high inoculation dose [28]. In 7-day-old challenged chickens, we observed a significant reduction of *Salmonella* spp. in the treated group at 7 dpi by culture and 14 dpi by qPCR analysis. Additionally, the number of positive chickens was significantly lower in the treated group at 7 dpi. These results may indicate that supplementation of poultry diet with FDA may delay and reduce *Salmonella* Typhimurium colonisation in the cecum of young broilers. A similar reduction of *S.* Typhimurium in the cecum of challenged broilers given supplements of fermented soybean has been reported [2].

The age of infection is known to influence pathogenesis in avian salmonellosis [31]. It is believed that a high dose is necessary to infect older chickens [28], although we maintained the same dose to allow comparison between the two challenge periods. Interestingly, we obtained higher values based on culture data for controls challenged at 21 days of life compared to 7-day-old controls, even if it is known that chickens over 3 weeks old are less susceptible to *Salmonella* spp. [31]. Moreover, we have observed a shortening time in the reduction of *Salmonella* spp. to low rates in 21-day-old challenged chicks compared to 7-day-old ones. In fact, it has been reported that 3-week-old broiler infection, as performed here, resulted in animals being infected for two weeks as the resolution of the *Salmonella* spp. infection occurred faster in old challenged chickens [26]. This could be partially explained by the increased immunity in older broilers as the age of infection seemed to influence *Salmonella* spp. persistence in the ceca [30]. Older animals have a more mature microbiota that, in our case, could provide colonization resistance and cause less colonization of pathogens, like *Salmonella.* In the treated group challenged at 21 days of life, we observed a reduction in the *Salmonella* spp. count by qPCR at 7 dpi, which is similar to what we observed in 7-day-old challenged chicks. This reduction was also confirmed by traditional culture, which reveals that FDA may also reduce *Salmonella* spp. colonisation in older newly-infected broilers. We agree with previous studies that infection, regardless of chicken age, resulted in the presence of *Salmonella* spp. in the intestine at slaughter age (42 days-old) [30]. Nevertheless, we observed a significant reduction of the *Salmonella* spp. carriage in the cecum of 21-day-old challenged animals of the treated group by qPCR at 42 days of life. Thus, FDA may help to reduce *Salmonella* spp. in the cecum of broilers and therefore contribute to diminishing carcass contamination at slaughter.

The effect of natural phenolic compounds has not been previously tested in an avian challenge model of *Salmonella* Typhimurium. The bioactive molecules like polyphenols contained in olive oil extracts are known to show marked antimicrobial activity against *Salmonella* spp. in concentrations as high as 5 × 10^5^ CFU/mL [12]. Even if their concentration in extracts is notably superior to those found in olive oil and their by-products [12], the synergistic action of the dialdehyde form of decarboxymethyl ligstroside, oleuropein aglycons, hydroxytyrosol, and tyroxol partially compensates this matter and retains antimicrobial proprieties [10]. The delaying and reduction of *Salmonella* Typhimurium colonisation in the cecum in the present study could be explained by the antimicrobial effects of phenols and polyphenols present in the compound tested [8,14]. Specifically, hydroxytyrosol has been reported to be more active against *Salmonella* spp. compared to oleuropein [13]. These effects on *Salmonella* spp. in vitro are bacteriostatic, inhibiting the division by reducing intracellular concentration of ATP and depolarising the cell membrane leading to bacterial death [8]. In addition, FDA also contains a significant proportion of fibre [14,15], which has been suggested to prevent pathogen adhesion to the intestinal surface [26].

Intestinal mucosa evaluation can provide information on health status in poultry as stressors such as enteric infections may lead to structural modifications [6,32,33]. We found similar histopathological features regardless of the age of infection in both the duodenum and cecum of broilers. These findings are in line with studies assessing *S*. Typhimurium-induced histopathology in chickens [29,34,35]. The inflammatory infiltrate observed was predominantly composed of lymphocytes and severely affected the duodenum. In fact, the duodenum has been reported to be intensively affected after a *Salmonella* spp. challenge, eliciting an immune response composed mainly of mononuclear cells (T-lymphocytes) and heterophils [36,37,38]. Furthermore, we found that supplementation with FDA slightly reduced the severity of the lesions in the treated group, which paralleled what has been reported after supplementation with arginine in the jejunum of broilers [35].

Many authors have reported shortening of villi after a *Salmonella* spp. challenge [2,6,7,29,35,39,40,41,42,43], which implies difficulty in absorption capacity and a reduction in body weight gain [29,40,41,44]. In our study, we were able to observe a significant increase in the duodenum villi height of the treated group in most samplings, as previously described in healthy broilers [15]. A similar increase in villus height in the jejunum of broilers given supplements of probiotics or prebiotics and challenged with *S.* Typhimurium has been reported [2,6,32,40,42,44,45]. Fermented soybean, butyric acid, sodium butyrate, oligosaccharides, zinc, and dietary clay supplementation in *S.* Typhimurium-challenged broilers resulted in an increase in villi height in the small intestine, mitigating *Salmonella* spp. colonisation effects [2,6,32,40,42,44]. Similarly, carvacrol essential oil has showed a protective effect in the villus structure against a *Campylobacter* spp. infection in broilers [45]. The increase in the height of the villi observed here contributes to enhancing the absorption capacity of the intestine in animals infected with *Salmonella* spp. [7,32], helping to palliate the functional compromise in digestion, transport, and absorption in the alimentary tract [42].

The immune system of young chicks is not fully competent until weeks after hatching [30], which is why they are more prone to developing systemic infections. Thus, unspecific immune response is important in pathogen clearance and response to damage in the intestine. In our study, duodenal and cecal crypts were deeper in the treated group in almost all samplings. It has been previously reported that FDA stimulates crypt growth in healthy broilers [15], thus safeguarding epithelial renewal. Epithelial turnover has shown to be fundamental to the response to insults to the superficial mucosa [14], as a rapid epithelial replication promotes a quick healing of superficial lesions. After *Salmonella* spp. infection, an increase in the depth of the crypts is expected to compensate superficial mucosal damage [6,29,41]. Intestinal microbiota contributes to host susceptibility to infections [43]. Sequencing data of the 16S rDNA showed that the population up to 21 days of life was composed mainly by *Enterobacteriaceae*, which drastically decreased thereafter [46,47]. *Enterobacteriaceae* has been reported as the most abundant bacterial family in *S*. Typhimurium-challenged broilers [35]. We observed a reduction in *Enterobacteriaceae* in treated groups in several samplings. Such reduction of *Enterobacteriaceae* in the chicken gut has been associated with an increase in small chain fatty acid production [47], so the reduction of *Enterobacteriaceae* after FDA consumption may, to some extent, stimulate small chain fatty acid production. It has been described that increased production of small chain fatty acid in the avian intestine may lead to an increase in epithelial renewal as well as in villi height [2]. Moreover, the small chain fatty acid butyrate has been reported to reduce *Salmonella* spp. colonisation in the cecum [44].

In 7-day-old challenged chickens, *Enterobacteriaceae* was replaced by *Lachnospiraceae* and *Ruminococcaceae* from 28 to 35 days of life as previously reported [47,48]. The functions of *Lachnospiraceae* and *Ruminococcaceae* involve the production of short chain fatty acids through the fermentation of indigestible polysaccharides [47,49]. In addition, *Lachnospiraceae* possess hydrolases that allow the digestion of starch and glycogen by means of the rupture of α-amylase bonds [47]. *Lachnospiraceae* and *Ruminococcaceae* have been reported to diminish their abundance in inflammatory processes due to reactive oxygen species production by inflammatory cells [49]. We observed a reduction in both families a week after infection in 7-day-old challenged chicks. However, in the 21-day-old challenge, we found an increase in both *Lachnospiraceae* and *Ruminococcaceae* in the treated group during the two weeks following the challenge. In 21-day-old challenged broilers, one week post-infection, *Lactobacillaceae* and *Ruminococcaceae* were the most abundant families in the treated group. *Lactobacillaceae* are carbohydrate fermenters, contributing to the production of short chain fatty acids in the poultry intestine [49], which suggests that there may be beneficial microbiota variations in the treated group. Moreover, *S*. Typhimurium infection is known to reduce *Lactobacillaceae* in the chicken gut [40], so fermented defatted ‘alperujo’ may also contribute to mitigating disbiosis in infected chickens.

## 5. Conclusions

Dietary supplementation with fermented defatted ‘alperujo’ (FDA) was, to some extent, effective in delaying and reducing *Salmonella* Typhimurium colonisation in the cecum after a 7- or 21-day-old challenge. The significant reduction observed in the cecum in 42-day-old broilers may suggest that FDA stimulates *Salmonella* Typhimurium clearance in the cecum and may contribute to diminishing the risk of carcass contamination at slaughter. Additionally, the improvement in mucosal integrity suggests that enhancing intestinal health helps to mitigate *Salmonella* spp. infection in the host, and production losses. Microbiota composition variations after supplementation may be beneficial and may help to prevent dysbiosis. These results could provide evidence that this olive oil by-product would contribute to prophylactic and therapeutic measures to reduce salmonellosis prevalence in poultry farms.

## Figures and Tables

**Table 1:** Fermented defatted “alperujo” (FDA) composition.

Determination	Results
Moisture 103° (%w.w.)	12.20
Crude protein (Kjeldahl) (%w.w.)	6.40
Brute fat (%w.w.)	3.00
Ash content (%w.w.)	7.70
Lignin (%w.w.)	23.30
Acid detergent fibre (%w.w.)	39.20
Neutral detergent fibre (%w.w.)	49.30
Tannins (%w.w.)	0.06
Oleic acidity index (%w.w.)	46.10
Peroxide value (%w.w.)	7.90
Total polyphenols (meq/kg)	0.89
Crude fibre (%w.w.)	27.70

**Table 2:** Results of culture (CFU/g) and qPCR (genome equivalent/g) in 7-day-old challenged chickens.

Days-Old		Control	Treated	*p*-Value ^1^
7	Culture	<10^2^	<10^2^	1.000
	qPCR	*	*	1.000
14	Culture	4.21 × 10^7^	<10^2^	0.008
	qPCR	9.94 × 10^5^	2.58 × 10^5^	0.056
21	Culture	5.40 × 10^7^	3.84 × 10^7^	0.381
	qPCR	9.95 × 10^5^	9.96 × 10^4^	0.032
28	Culture	9.78 × 10^5^	2.93 × 10^5^	1.000
	qPCR	3.53 × 10^5^	3.86 × 10^5^	1.000
35	Culture	5.23 × 10^5^	2.19 × 10^5^	1.000
	qPCR	2.25 × 10^3^	6.95 × 10^3^	0.222
42	Culture	<10^2^	<10^2^	1.000
	qPCR	3.14 × 10^4^	*	0.444

^1^ The Mann–Whitney test was used to assess significant differences between controls and animals given supplements (*p* < 0.05). * Value under the detection limit (one *Salmonella* spp. genome equivalent/reaction) [20].

**Table 3:** Results of culture (CFU/g) and qPCR (genome equivalent/g) in 21-day-old challenged chickens.

Days-Old		Control	Treated	*p*-Value ^1^
7	Culture	<10^2^	<10^2^	1.000
	qPCR	*	*	1.000
14	Culture	<10^2^	<10^2^	1.000
	qPCR	*	*	1.000
21	Culture	<10^2^	<10^2^	1.000
	qPCR	*	*	1.000
28	Culture	7.77 × 10^7^	2.51 × 10^7^	0.075
	qPCR	2.37 × 10^6^	4.68 × 10^5^	0.016
35	Culture	1.49 × 10^7^	1.02 × 10^5^	0.602
	qPCR	8.04 × 10^5^	4.95 × 10^5^	0.310
42	Culture	7.37 × 10^6^	1.35 × 10^6^	0.076
	qPCR	5.18 × 10^5^	1.31 × 10^5^	0.016

^1^ The Mann–Whitney test was used to assess significant differences between controls and animals given supplements (*p* < 0.05). * Value under the detection limit (one *Salmonella* spp. genome equivalent/reaction) [20].

**Table 4:** Number of animals testing positive for *Salmonella* spp. by culture and qPCR.

		7-Day-Old Challenged			21-Day-Old Challenged		
Days-Old		Control	Treated	*p*-Value ^1^	Control	Treated	*p*-Value ^1^
7	Culture	0/5	0/5	^2^	0/5	0/5	^2^
	qPCR	0/5	0/5	^2^	0/5	0/5	^2^
14	Culture	5/5	0/5	>0.05	0/5	0/5	^2^
	qPCR	5/5	4/5	>0.05	0/5	0/5	^2^
21	Culture	5/5	3/5	>0.05	0/5	0/5	^2^
	qPCR	5/5	4/5	>0.05	0/5	0/5	^2^
28	Culture	3/5	3/5	>0.05	5/5	3/5	>0.05
	qPCR	5/5	5/5	^2^	5/5	5/5	^2^
35	Culture	2/5	2/5	>0.05	5/5	5/5	^2^
	qPCR	5/5	5/5	>0.05	5/5	5/5	^2^
42	Culture	0/5	0/5	^2^	5/5	5/5	^2^
	qPCR	2/5	0/5	>0.05	5/5	5/5	^2^

^1^ The Fisher’s exact test was used to assess significant differences between control and treated animals. ^2^ No statistics are computed because the value obtained is a constant.

**Table 5:** Histomorphometric results: duodenum villi height, duodenum crypt depth and ceca crypt depth. Number of samples (N), mean values (mean), standard deviation (SD), and *p*-value are detailed per day of life and challenge group.

	Control			Supplemented			*p*-Value ^1^
	N	Mean	SD	N	Mean	SD	
**7-Day-Old Challenge**							
Duodenum Villus Heights							
7 days	100	539.81	192.04	100	853.62	217.81	<0.001
14 days	100	896.95	193.71	100	1028.75	398.60	0.006
21 days	100	1208.45	281.81	100	1188.42	202.38	0.767
28 days	100	1118.62	225.74	100	1200.56	246.92	0.011
35 days	100	893.23	203.78	100	1258.92	269.51	<0.001
42 days	100	923.01	225.90	100	1310.98	241.50	<0.001
Duodenum Crypt Depths							
7 days	100	78.14	16.49	100	89.16	26.49	0.001
14 days	100	93.91	29.84	100	131.74	134.48	<0.001
21 days	100	128.09	49.01	100	141.61	44.84	0.005
28 days	100	137.74	39.52	100	153.28	35.11	0.001
35 days	100	124.56	37.50	100	151.71	38.22	<0.001
42 days	100	107.82	37.22	100	122.56	39.46	0.012
Cecum Crypt Depths							
7 days	100	181.72	61.43	100	227.93	105.39	0.002
14 days	100	266.79	76.54	100	288.37	90.69	0.189
21 days	100	204.25	71.28	100	235.64	91.31	0.028
28 days	100	276.78	69.49	100	266.57	109.68	0.006
35 days	100	234.47	65.44	100	249.76	78.30	0.208
42 days	100	233.19	67.96	100	353.70	165.79	<0.001
**21-Day-Old Challenge**							
Duodenum Villus Heights							
28 days	100	1052.40	323.90	100	1353.79	220.63	<0.001
35 days	100	1039.11	233.53	100	1193.80	195.27	<0.001
42 days	100	888.01	200.58	100	1210.86	233.54	<0.001
Duodenum Crypt Depths							
28 days	100	145.07	45.43	100	158.67	47.70	0.033
35 days	100	125.94	35.36	100	133.36	33.81	0.179
42 days	100	120.93	32.62	100	154.44	36.30	<0.001
Cecum Crypt Depths							
28 days	100	298.80	144.55	100	272.91	116.46	0.354
35 days	100	230.23	128.98	100	325.47	236.05	<0.001
42 days	100	238.19	98.04	100	409.40	246.84	<0.001

^1^ The Mann–Whitney test was used to assess significant differences between controls and animals given supplements (*p* < 0.05).
